# Development of a questionnaire to assess interprofessional collaboration between two different care levels

**DOI:** 10.5334/ijic.984

**Published:** 2013-04-12

**Authors:** Roberto Nuño-Solinís, Iñaki Berraondo Zabalegui, Regina Sauto Arce, Leticia San Martín Rodríguez, Nuria Toro Polanco

**Affiliations:** O+Berri – Basque Institute for Healthcare Innovation, Plaza de Asua 1, 48950 Sondika (Bizkaia), Spain, Kronikgune Research Group on Population Health Management; Bidasoa Integrated Healthcare Organisation, Finca Zubieta s/n, Hondarribia (Gipuzkoa), Spain, Kronikgune Research Group on Action-Research on Integrated Care; O+Berri – Basque Institute for Healthcare Innovation, Plaza de Asua 1, 48950 Sondika (Bizkaia), Spain, Kronikgune Research Group on Population Health Management; Hospital University of Navarra, Avenida Pío XII 36, Pamplona, Spain, Kronikgune Research Group on Population Health Management; O+Berri – Basque Institute for Healthcare Innovation, Plaza de Asua 1, 48950 Sondika (Bizkaia), Spain, Kronikgune Research Group on Population Health Management

**Keywords:** health services research, interprofessional relations, questionnaires, validation studies

## Abstract

**Introduction:**

This paper reports the development and validation of a questionnaire to assess collaboration between clinical professionals from two different care levels (primary and specialised care), according to the clinicians’ own perceptions. This questionnaire has been elaborated to be used as part of the monitoring and evaluation process of the integrated care pilots in the public Basque Health Service.

**Methods:**

The process was carried out in four phases: development of the first version of the questionnaire, validation of the content, pre-testing, and evaluation of its construct validity and homogeneity in a sample of doctors and nurses. This last phase involved confirmatory factor analysis, as well as the calculation of Cronbach’s α and various correlation coefficients.

**Results:**

The process demonstrated that the theoretical content of the questionnaire was appropriate, and also that its items were clear, relevant and intelligible. The fit indices for the confirmatory factor analysis were: χ^2^ of 45.51 (p=0.089), RMSEA of 0.043, RMR of 0.046, GFI of 0.92 and CFI of 0.99.

**Discussion:**

The statistics indicate a good fit between the data and a conceptual two-factor structure, in which both personal relationships between professionals and characteristics of the organisational environment are understood to underlie interprofessional collaboration.

**Conclusion:**

The end-product is a new instrument with good validity to assess the degree of interprofessional collaboration between clinicians working at two different levels of care.

## Introduction

In recent years, a range of initiatives of integration have been launched within the Basque Health Service (Osakidetza), in response to new policies for transforming the system towards better prevention and care for chronic diseases. These policies are set out in a strategic document entitled “A Strategy to Tackle the Challenge of Chronicity in the Basque Country” [1], published by the Department of Health and Consumer Affairs of the Basque Government in July 2010. In this document, the Basque health authorities highlighted the need to improve the integration and continuity of care for patients with chronic diseases, as one of five priority areas for action to address the challenge of chronicity.

Since then, a number of integration initiatives have sprung up in the public Basque Health Service, which can be broadly classified into three main types. On the one hand, there are initiatives of organisational integration, particularly the creation of new integrated delivery organisations. These organisations merge previously separated healthcare settings—generally a regional hospital (specialised care level) and the health centres (primary care level) of the area around the hospital—under a single management structure and a common contracting and financial framework, which would jointly serve the population under their responsibility. On the other hand, a range of initiatives that can be broadly defined as disease management programmes have been emerging across the Basque health service, aiming at integrating care processes, while respecting the organisational separation between care levels. This type of initiatives focuses on specific conditions and groups of patients. Several of these initiatives include the use of tele-monitoring tools and the roles of case managers and link nurses. For example, in a project called PROMIC, a multidisciplinary team including primary and specialised care professionals working in different organisations, has been established, and case managers introduced, to coordinate and control care of high-risk patients with heart failure and co-morbidities, for whom a common care pathway has been agreed. Finally, a third type of integration initiatives, which could be defined as shared care models for patients with multiple conditions and complex needs, can be distinguished. In the Basque case, initiatives targeting these patients at the top of the Kaiser pyramid [2], include the identification of a team or a specialist of reference for the primary care team and the complex patient at the hospital, and often involve the use of case managers. All these different types of integration initiatives are mostly still in the pilot phase, having had until now an impact on only a limited number of healthcare settings and certain specific units and services. All of them require collaboration between professionals from different care levels and specialties, most often working in different healthcare settings and/or organisations.

Due to the interest on monitoring and assessing the results of these integration initiatives, an overarching evaluation framework for integrated care pilots in the public Basque Health Service was developed (and published elsewhere [3]). Within this broader evaluation framework, a culture that favours interprofessional collaboration between different care levels and settings was considered a key element for improving coordination of services and continuity of care [4, 5], and as such, identified as an important dimension to be monitored. So, it was deemed necessary to identify a measure of how interprofessional collaboration, as a core factor for integrated care [6], changed with time and with the development of the different types of integration initiatives in place.

The objective of this article is to describe the process of development and validation of a questionnaire that was produced in response to this need to evaluate interprofessional collaboration between different care levels. This questionnaire, which is based on the perceptions by the clinicians concerned (and initially validated in a group of doctors and nurses), is currently being used as part of the broader monitoring and evaluation process of several of the healthcare integration pilots in the Basque Health Service [3].

## Methods

**Figure 1.**
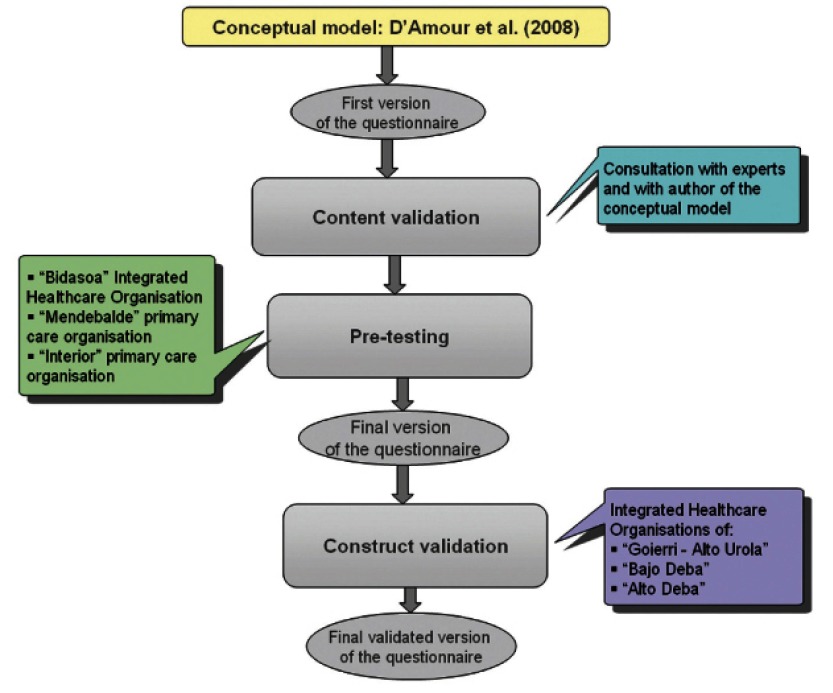
Process of designing and validating the questionnaire.

The process of developing and validating the questionnaire was carried out in four phases: 1) development of the first version of the questionnaire, 2) validation of the content, 3) pre-testing, and 4) evaluation of its construct validity and homogeneity. Figure 1 illustrates the different parts of the process.

### Development of the first version of the questionnaire

A first important step was choosing the theoretical framework of reference as regards interprofessional collaboration between different care levels and healthcare settings/organisations. A previous literature review was used for the identification of the most complete conceptual frameworks of interprofessional collaboration in the health field, according to the criteria established by the authors of the review [7]. From this literature review, three frameworks were pre-selected: the models by Sicotte et al. [6], West et al. [8], and D’Amour et al. [9]. All these three models fulfilled the criteria of being based both on empirical data and on an explicit theory, and were examined by the authors. Finally, a choice was made for the model by D’Amour et al. [9, 10], as the one best suited to the objectives of the evaluation and the organisational context in the Basque health service. In fact, this was the only among the three pre-selected models that focused on interorganisational collaboration, which adjusted to the interest of the authors on interprofessional collaboration across care levels and between different healthcare settings. It also made an explicit link between interorganisational collaboration and continuity of care for patients, what accommodated the authors’ concern about interprofessional collaboration as an intermediate outcome on the achievement of further objectives of integrated care, such as better coordination and continuity of care for patients [3].

**Figure 2.**
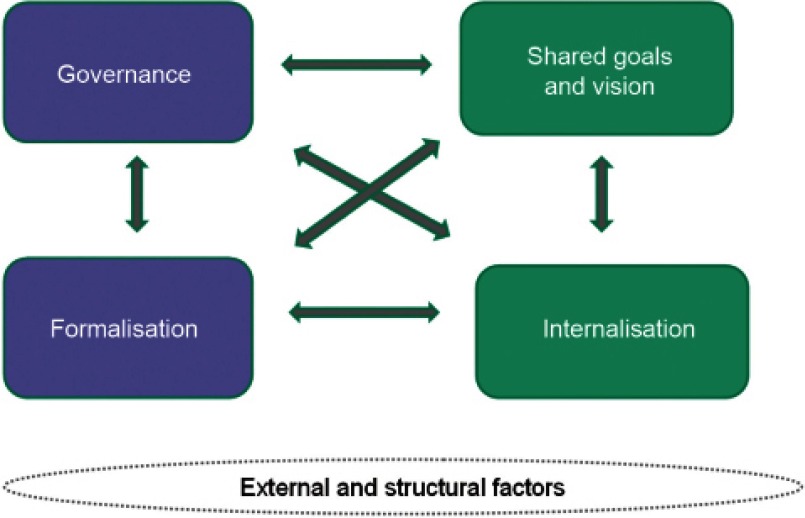
D’Amour’s dimensions of collaboration between health professionals and organisations [13].

The D’Amour model is inspired by the concept of collective action in organisational sociology, in particular, strategic analysis as in Crozier and Friedberg [11] and the organisational approach proposed by Friedberg [12]. According to D’Amour, collaboration is the structuring of collective action through the sharing of information and decision-making in clinical processes. The model identifies four dimensions that characterise the processes of interprofessional collaboration, two related to relationships between individuals (shared goals and vision; and internalisation) and two related to the organisational setting (governance; and formalisation) (Figure 2). All these dimensions are interrelated and present in all collective action. The intensity and impact of each, however, depends on the specific situation and context. D’Amour also recognises that other external and structural factors may influence interprofessional collaboration.

This model has been operationalised by D’Amour and colleagues [13] through the identification of ten indicators, validated in Québec (Canada), of the collaboration between professionals in different healthcare organisations. These indicators are listed in Table 1 for each of the four dimensions.

Taking these four dimensions and ten indicators (as defined in D’Amour et al. [13]) as a point of departure, a first version of the questionnaire was drawn. It included ten items, one for each of the aforementioned indicators. Response options for each item consisted of a 5-point Likert scale, where one corresponded to none and five to a high degree of development. Initially only these extreme ratings were anchored by descriptive phrases.

### Validation of the content

The initial version of the questionnaire was sent for consultation to five Spanish experts in care integration identified by consensus among the authors, as well as to the developer of the conceptual model behind it, the Canadian Danielle D’Amour. Their comments led to modifications both in content and format, though the 10-item structure and the 5-point Likert scale were maintained. Specifically, the wording of the items and of the response options was changed, in order to more clearly limit the scope of each item to a single aspect of interprofessional collaboration. Moreover, a description of the degree of development corresponding to the mid-point of the scale (3 on the Likert scale) was added.

### Pre-testing

The following phase was to pre-test the questionnaire in three healthcare organisations in the Basque health service, with the purpose of assessing its intelligibility, clarity and relevance, as well as the time required to complete it. A total of 24 clinical professionals (doctors and nurses) gave their opinion on the intelligibility, clarity, and relevance of the items and the response options, some in a face-to-face meeting and others in writing. Their views enabled the authors to fine-tune the instrument: 1) the wording of the second item was slightly changed; 2) implied value judgements, which could potentially bias responses, were eliminated from the items and response options; 3) extra descriptions were added for response options so that, in the end, descriptive phrases for all five points on the Likert scale were provided; and 4) modifications were made to move, as far as possible, towards a uniform style for all items and response options. As a result, a new—final—version of the questionnaire was elaborated, which is provided in the Appendix (original version in Spanish). For an international audience, a direct translation of the questionnaire into English is also included in Appendix.

### Evaluation of the construct validity and homogeneity

The last phase of the process consisted on assessing the construct validity and homogeneity of the items in the final version of the instrument.

#### Sample

The sample comprised 187 clinical professionals (doctors and nurses) working in three integrated healthcare organisations in the Basque Health Service (‘Goierri-Alto Urola’, ‘Alto Deba’ and ‘Bajo Deba’). Regarding the characteristics of the professionals in the sample: 43% were primary care nurses, 31% primary care doctors (GP or paediatrician), 18.5% hospital specialists and 6% hospital nurses; their average age was 45 years (standard deviation: 8); and 23% were men.

#### Data collection

A link to an electronic version of the questionnaire was sent by e-mail, via the managerial team of each of the three integrated healthcare organisations involved in the validation, to all the doctors and nurses in their organisations (this included a total of 1166 professionals, of which 564 doctors and 602 nurses). This version was created using ‘Google Docs’ and could be completed online or directly from email. Responses were collected during February and March 2012.

#### Data analysis

On the one hand, to assess the construct validity of the Spanish version of the instrument, a first exploratory and then confirmatory factor analysis were conducted, with SPSS 15.0 and LISREL 8.80 software, respectively. Exploratory analysis was carried out using principal component analysis with a Promax type oblique rotation. Potential factors were assessed in the light of common objectives, namely that eigenvalues were >1 and explained more than 5% of the variance [14, 15]. The confirmatory factor analysis then served to assess the goodness-of-fit of the proposed conceptual structure, considering the data from the exploratory analysis and the original model. Given the ordinal nature of the data, this analysis was conducted using polychoric correlation and asymptotic variance-covariance matrices [16–19]. The goodness-of-fit was assessed using a weighted least squares approach, as suggested by Jöreskog for ordinal data [18]. The overall fit to the conceptual model was assessed using a set of indices [20, 21]. These indices are listed in Table 2, along with the corresponding threshold levels for a good fit.

On the other hand, to assess the reliability of the instrument, the homogeneity of the items was explored. For this, Cronbach’s α coefficient was calculated for the items comprising each factor as well as for all the items in the questionnaire. Further, adjusted item total score correlation coefficients were calculated. All this analysis was performed with SPSS 15.0 software.

## Results

Both the Kaiser-Meyer-Olkin measure of sampling adequacy (0.916) and Bartlett’s test of sphericity (p<0.001) confirmed a strong enough relationship between the items in the correlation matrix to justify factor analysis.

First, the exploratory analysis identified a component that explained 49.9% of the variance and provided evidence to support the idea that the instrument would be best represented by a two-factor structure. The eigenvalue of the second component was slightly less than but close to 1 (0.938) and explained 9.38% of the variance. The rotation to facilitate interpretation of the results was carried out with all items, given that the contribution of each of them to the instrument, as well as the loading, was higher than 0.4, in all factors [22]. After rotation, none of the items was eliminated, given that the differences in saturation between factors was higher than |0, 10| in all cases. Table 3 reports the final results after rotation.

Taking into account that the conceptual model underlying the instrument holds that there are two major dimensions within the concept of collaboration (personal relationships and the organisational setting), a confirmatory factor analysis was performed to test the two-factor structure of this theoretical model against the data.

**Figure 3.**
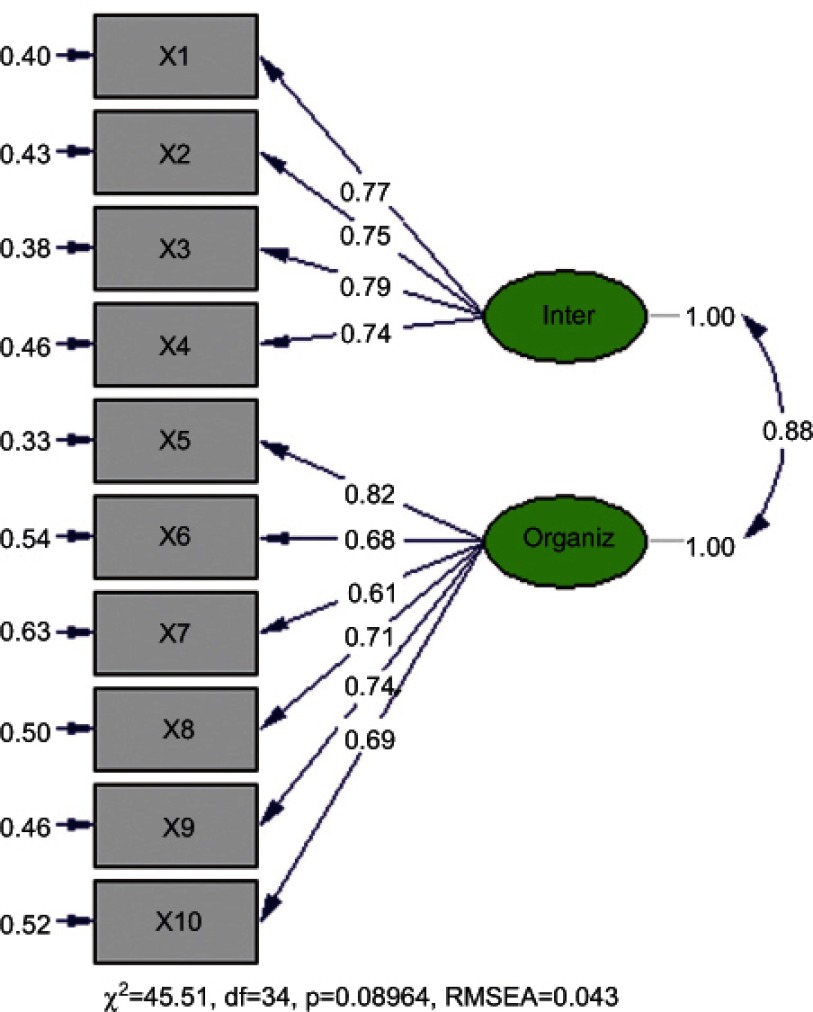
Results of the confirmatory factor analysis.

The confirmatory factor analysis of the two-factor structure yielded values of 0.61 to 0.82 for the parameters, indicating the link between each item and its corresponding factor. The estimated values for each of the parameters and the corresponding standard errors (between 0.33 and 0.63) are shown on Figure 3 for each of the ten items on the questionnaire. The results of the confirmatory factor analysis also show that the coefficients of determination (R^2^) for all 10 items vary in a range between 0.37 and 0.67. These coefficients represent the percentage of the systemic variance of each item explained by the model.

As shown in Figure 3, there is high correlation (88% of the maximum possible correlation) between the two factors of the model.

Regarding internal consistency, a Cronbach’s α coefficient of 0.866 for the 10 items was obtained, and, per factor, of 0.813 for the personal relationships dimension and 0.825 for the organisational setting dimension. The adjusted item total score correlation coefficients ranged from 0.548 to 0.676. Table 4 reports the results of this analysis in more detail.

## Discussion

Having first selected a theoretical model to conceptualise the phenomenon of collaboration between professionals at different levels of care and converted the ten indicators proposed by the developer of the model [13] into items, the validation of the content and pre-test of the questionnaire were carried out. Fine tuning of the questionnaire was based on feedback from a group of experts, including the developer of the conceptual model, and from a group of clinical professionals who assessed the first version, as for the suitability of the content from a theoretical point of view, as well as the clarity, relevance and intelligibility of the items that make up the questionnaire.

Results of the analysis of the construct validity, during the validation phase, indicate that there is a good match between the instrument and the underlying conceptual model. Specifically, confirmatory factor analysis, following exploratory analysis to identify potential factors, shows that the questionnaire developed has a two-factor structure, reflecting the conceptual model on which it was based. The fit indices support this assertion. This conceptual structure proposes that there are two aspects or main dimensions of collaboration: one related to interpersonal relationships between professionals and the other to characteristics of the organisational environment. The personal relationships dimension is related to the existence of shared goals, a patient-centred focus, mutual knowledge and trust, while the organisational setting dimension refers to the degree of centrality, leadership, connectivity, support for innovation, formalisation tools and information exchange. Five of the ten estimated parameters for the items had significance values of <0.05 and the other five values <0.10, reflecting the strength of the association between the items and the corresponding dimension.

The fact that, on the one hand, the indices show an adequate adjustment to the bi-factorial structure, and on the other hand, there is a conceptual meaning to the two main factors (an interpersonal and an organisational dimension), has been decisive for keeping the two factors, despite the high correlation between them.

Further, the results indicate that the questionnaire has good homogeneity. In particular, the Cronbach α coefficients were over 0.80, exceeding the threshold of 0.70 proposed by Nunnally [23]. On the basis of these values, it can be stated that 88.6% of the variance is systematic, that is, this percentage of the variance represents the actual differences between individuals in terms of their perception of the degree of collaboration, while the rest (11.4%) is attributable to random variations [14, 24, 25].

As for the adjusted item-total score correlation coefficients, reflecting the contribution of each item to the total score, the values were considerably over the 0.3 threshold established by Ebel and Frisbie for this type of correlation [26]. Specifically, the coefficients were higher than 0.5 for all of the items, more than half of them (6/10) being higher than 0.6.

It should be noted, that the reliability of a measure is not a property of an instrument itself, but rather of an instrument administered in a specific sample under certain conditions [25], in our case, a group of doctors and nurses in three integrated healthcare organisations in the public Basque Health Service. Accordingly, it is proposed that the two-factor structure is further tested in other studies using different samples. Further, to be able to generalise the results, it would be interesting for the overall psychometric properties of the instrument to be assessed using samples from other groups of healthcare professionals and organisational contexts. In addition, it would be interesting to validate a translated version of this Spanish questionnaire.

Among the methodological limitations of this analysis, one could mention the fact that the same data set was used both for the exploratory and for the confirmatory factor analysis. In addition, this study analysed the internal consistency and homogeneity of the developed instrument, but other psychometric properties, such as stability (through, for example, a test-retest) or convergent validity, were not tested.

## Conclusion

As the end-product of this project, a Spanish version of a questionnaire to measure interprofessional collaboration between clinical professionals at different levels of care (primary and specialised care), based on the conceptual model developed by D’Amour and colleagues [27], has been produced. This questionnaire can be considered to have good validity to measure degree of collaboration between clinical professionals from different care levels. The various types of analysis undertaken indicate that the instrument has a two-factor structure, in which collaboration is understood to involve characteristics of the interpersonal relationships between professionals and of organisations themselves. The questionnaire has also been shown to have good internal consistency and homogeneity in the group of doctors and nurses from three integrated healthcare organisations of the Basque health service, where it was administered.

## Acknowledgements

The authors are grateful for the support received from Danielle D’Amour, from the Spanish experts whose opinion was sought, as well as from the six healthcare organisations within the public Basque Health Service (Osakidetza) that participated in the validation of the questionnaires (Bidasoa Integrated Healthcare Organisation, Interior Primary Care Organisation, Mendebalde Primary Care Organisation, Alto Deba Integrated Healthcare Organisation, Bajo Deba Integrated Healthcare Organisation and Goierri-Alto Urola Integrated Healthcare Organisation).

## Reviewers

**Manuel García Goñi,** Associate Professor of Applied Economics, Universidad Complutense de Madrid, Spain

**Angus McFadyen,** Statistical Consultant, AKM-STATS, Glasgow, Scotland

One anonymous reviewer.

## Figures and Tables

**Table 1.**
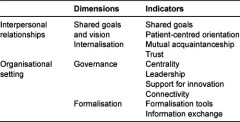
Dimensions and corresponding indicators of the conceptual model used as a basis for the questionnaire.

**Table 2.**
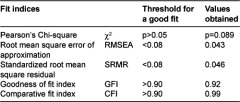
Fit indices.

**Table 3.**
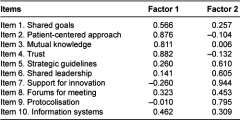
Matrix with final exploratory factor analysis.

**Table 4.**
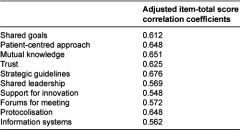
Adjusted item-total score correlation coefficients.

## Appendix

### Appendix 1: Questionnaire—Spanish version

Cuestionario de valoración de la colaboración entre profesionales clínicos de distintos niveles de atención

### Appendix 2: Questionnaire—English translation

Questionnaire to assess interprofessional collaboration between two different care levels
